# Microcirculatory alterations: potential mechanisms and implications for therapy

**DOI:** 10.1186/2110-5820-1-27

**Published:** 2011-07-19

**Authors:** Daniel De Backer, Katia Donadello, Fabio Silvio Taccone, Gustavo Ospina-Tascon, Diamantino Salgado, Jean-Louis Vincent

**Affiliations:** 1 Department of Intensive Care, Erasme University Hospital, Université Libre de Bruxelles, Route de Lennik 808, B-1070 Brussels, Belgium

## Abstract

Multiple experimental and human trials have shown that microcirculatory alterations are frequent in sepsis. In this review, we discuss the characteristics of these alterations, the various mechanisms potentially involved, and the implications for therapy. Sepsis-induced microvascular alterations are characterized by a decrease in capillary density with an increased number of stopped-flow and intermittent-flow capillaries, in close vicinity to well-perfused capillaries. Accordingly, the surface available for exchange is decreased but also is highly heterogeneous. Multiple mechanisms may contribute to these alterations, including endothelial dysfunction, impaired inter-cell communication, altered glycocalyx, adhesion and rolling of white blood cells and platelets, and altered red blood cell deformability. Given the heterogeneous nature of these alterations and the mechanisms potentially involved, classical hemodynamic interventions, such as fluids, red blood cell transfusions, vasopressors, and inotropic agents, have only a limited impact, and the microcirculatory changes often persist after resuscitation. Nevertheless, fluids seem to improve the microcirculation in the early phase of sepsis and dobutamine also can improve the microcirculation, although the magnitude of this effect varies considerably among patients. Finally, maintaining a sufficient perfusion pressure seems to positively influence the microcirculation; however, which mean arterial pressure levels should be targeted remains controversial. Some trials using vasodilating agents, especially nitroglycerin, showed promising initial results but they were challenged in other trials, so it is difficult to recommend the use of these agents in current practice. Other agents can markedly improve the microcirculation, including activated protein C and antithrombin, vitamin C, or steroids. In conclusion, microcirculatory alterations may play an important role in the development of sepsis-related organ dysfunction. At this stage, therapies to target microcirculation specifically are still being investigated.

## Introduction

Sepsis is associated with high mortality. Multiple mechanisms may contribute to sepsis-associated organ dysfunction, which is related to altered tissue perfusion, especially in the early stages, and to direct alterations in cellular metabolism. The importance of rapid correction of perfusion abnormalities has lead to the concept of early goal-directed therapy, which has been shown to improve the outcome of patients with septic shock [1]. However, even when global hemodynamics are optimized, alterations in the microcirculation can still be present and can contribute to perfusion alterations [2]. Indeed, the microcirculation is responsible for fine-tuning tissue perfusion and adapting it to metabolic demand. Experimental and, more recently with development of new techniques that allow direct visualization of the microcirculation [3], clinical evidence indicate that microcirculatory alterations occur in severe sepsis and septic shock and that these alterations may play a role in the development of organ dysfunction. In this review, we will discuss the relevance of these sepsis-associated microcirculatory alterations, the mechanisms involved in their development and potential therapies.

### Methods to evaluate microcirculation in humans

Several methods can be used to evaluate microcirculation in septic patients [3]. Two techniques are currently used to evaluate microcirculation at bedside: Sidestream Sark Field imaging technique (SDF) and near infrared spectroscopy (NIRS). SDF is a small handheld microscope that illuminates the field by light reflection from deeper layers. Vessels are visualized as the selected wavelength is absorbed by the hemoglobin contained in the red blood cells.

Orthogonal Polarization Spectral imaging technique (OPS) was based on a similar principle but is no longer available. The technique is limited by the fact that it can only be applied on superficial tissues covered by a thin epithelium (mostly the sublingual area) and it requires collaboration or sedation of the patient. In addition, great care should be taken to discard secretions and to limit pressure artifacts.

NIRS utilizes near-infrared light to measure oxy- and deoxy-hemoglobin in tissues and to calculate StO2 (tissue oxygen saturation, measured by NIRS in thenar eminence). In fact, StO2 represents the oxygen saturations of all vessels with a diameter less than 1 mm (arterioles, capillaries, and venules) comprised in the sampling volume, with venules accounting for 75% of the blood volume. Basal StO2 is of limited interest, because there is a huge overlap between StO2 values obtained in septic patients and in intensive care unit (ICU) controls or healthy volunteers. StO2 also differs from central venous O2 saturation (ScvO2) in sepsis. The analysis of changes in StO2 during a brief episode of forearm ischemia enables quantification of microvascular reserve. Several indices can be measured, but the ascending slope, or recovery slope, is the easiest to measure and is the most reproducible. At the present time, both SDF and NIRS are mostly used for research purposes.

### Microcirculatory alterations are observed in severe sepsis

Multiple investigations in various experimental models have shown that sepsis is associated with a decrease in capillary density in association with increased heterogeneous perfusion in visualized capillaries, such that capillaries with intermittent or no flow are found in close proximity to well-perfused capillaries [4-7]. Importantly, capillaries in which there is no flow at a given time may be well perfused a few minutes later, and perfused vessels may later have no flow. The microcirculation is a very dynamic process, and space and time heterogeneity are increased in septic conditions. These alterations have been observed in different models of sepsis, including those created by administration of endotoxin or live bacteria and bacterial peritonitis [5,6,8], in all organs investigated, including the skin, tongue [6], gut [6,7], liver [4], and even the brain [8], and in all species that have been investigated, from rodents [5,9] to large animals [6,8]. Hence, these changes seem to be ubiquitous and to have common pathophysiologic mechanisms.

In patients with severe sepsis and septic shock, we first demonstrated that microcirculatory perfusion is altered in a similar way to that occurring in experimental conditions [2]. Compared with healthy volunteers and ICU controls, patients with severe sepsis have a decrease in vascular density together with an increased number of capillaries with stopped or intermittent flow. Importantly, these alterations can be fully reversed by topical application of acetylcholine, indicating that microthrombi are not an essential component. Since this early study, more than 25 studies from different teams around the world have shown similar results (Table 1).

**Table 1:** Studies that have reported alterations in sublingual microcirculation in patients with severe sepsis and septic shock

Reference	No. of patients	Intervention
De Backer et al. AJRCCM 2002	50	Topical acetylcholine
Spronk et al. Lancet 2002	6	Nitroglycerin
Sakr et al. CCM 2004	49	Sequential assessment
De Backer et al. CCM 2006	22	Dobutamine
De Backer et al. CCM 2006	40	Activated protein C
Creteur et al. ICM 2006	18	Dobutamine
Boerma et al. CCM 2007	23	Sequential assessment
Trzeciak et al. Ann Emerg Med 2007	26	None
Sakr et al. CCM 2007	35	Transfusions
Trzeciak et al. ICM 2008	33	Goal directed therapy
Boerma et al. ICM 2008	35	None
Jhanji et al. ICM 2009	16	Norepinephrine
Dubin et al. Crit Care 2009	20	Norepinephrine
Buchele et al. CCM 2009	20	Hydrocortisone
Boerma et al. CCM 2010	70	Nitroglycerin
Ospina et al. ICM 2010	60	Fluids
Spanos et al. Shock 2010	48	None
Pottecher et al. ICM 2010	25	Fluids
Morelli et al. Crit Care 2010	40	Levosimendan
Ruiz et al. Crit Care 2010	12	High flow hemofiltration
Dubin et al. J Crit Care 2010	20	Fluids
Morelli et al. ICM 2011	20	Terlipressin

### Relevance of sepsis-associated microcirculatory alterations

Because the microcirculation is essentially adaptive, it is important to understand whether the sepsis-associated alterations are the primary event leading to cellular dysfunction or whether the changes in perfusion reflect directly altered cellular metabolism (adaptive theory). In experimental conditions, it has been possible to link microvascular impairment to signs of tissue hypoxia: colocalization of low PO_2_, production of hypoxia inducible factor [10] or redox potential [11] with hypoperfused vessels suggest that the altered perfusion leads to tissue dysoxia and not the reverse. In addition, oxygen saturation at the capillary end of well-perfused capillaries is low, suggesting that the tissues are using the delivered oxygen.

In septic patients, microcirculatory alterations are more severe in nonsurvivors than in survivors [2]. By sequentially assessing the sublingual microcirculation in patients with septic shock, Sakr et al. [12] observed that the microcirculation is rapidly improved in survivors, whereas in nonsurvivors it remained disturbed, whether these patients died from acute circulatory failure or later, after resolution of shock, from organ failure. Similar results were recently observed in children with septic shock [13]. Trzeciak et al. [14] also observed that early (within 3 h) improvement of sublingual microcirculation in response to resuscitation procedures was associated with an improvement of organ function at 24 h, whereas patients whose microcirculation did not improve experienced a worsening of organ function.

### Mechanisms involved in the regulation of microcirculatory perfusion in normal conditions

Tissue perfusion is determined by vascular density--the diffusive component--and by flow--the convective component. Capillary density increases in response to chronic hypoxia [15] or during training [16]. In exercise, the maximal oxygen consumption is proportional to muscle capillary density. However, this adaptative process may take several weeks to occur. In more acute situations, there is a small reserve for capillary recruitment, mostly because a few capillaries are shut down at baseline. Compared with baseline, the heterogeneity of the microcirculation increased by close to 10% during hypoxia or hemorrhage [7].

How do capillary flow and density adapt in normal conditions? In healthy conditions, the microcirculation is responsible for fine-tuning of perfusion to meet local oxygen requirements. This is achieved by recruiting and derecruiting capillaries, shutting down or limiting flow in capillaries that are perfusing areas with low oxygen requirements and increasing flow in areas with high oxygen requirements. This process implies local control of flow, which needs to be driven by backward communication. Indeed, release of vasoactive or hormonal substances can only lead to downstream adaptation, but it is upstream adaptation that is required. Two mechanisms may help with this local communication: perivascular sympathetic nerves [17], which mostly influence the control of arteriolar tone, and backward communication along the endothelium, mediated by endothelial cells themselves. In addition, red blood cells may act as intravascular sensors [18]. The decrease in oxygen saturation that occurs as a result of oxygen offloading causes the local release of nitric oxide, leading to capillary dilation at the site where it is needed.

What drives blood flow in the capillaries? According to Poiseuille's law, flow in a capillary is proportional to the driving pressure (ΔP) and to the fourth power of the capillary radius (r), and inversely proportional to capillary length (L) and blood viscosity (η):

Because capillary length and viscosity cannot be actively manipulated, capillary flow can only be adapted by local dilation and increased driving pressure. Because capillaries are situated downstream of resistive arterioles, an increase in driving pressure can only be obtained by vasodilation of resistive arterioles. Hence, in normal conditions, the organism is continuously fine-tuning microvascular density and flow by subtle dilation/constriction of selected arterioles and capillaries.

Importantly, it should be remembered that capillary hematocrit is less than systemic hematocrit, due to the necessary presence of a plasma layer at the endothelial surface. Accordingly, hematocrit is proportional to capillary radius, so vasodilation will markedly increase local oxygen delivery as the result of a combined increase in flow and in oxygen content.

Finally, it should be noted that adaptation of capillary perfusion at the organ level does not depend on systemic arterial pressure and cardiac output but, of course, will result in increased cardiac output if venous return increases as a result of a major increase in capillary flow, as during exercise or feeding.

### Mechanisms that may be involved in the development of microcirculatory alterations in sepsis

**Figure 1:**
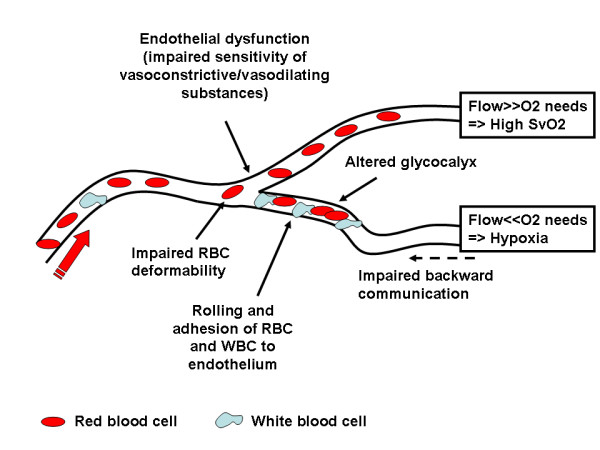
**Principal mechanisms implicated in the development of microcirculatory alterations**.

Several mechanisms are implicated, including endothelial dysfunction, altered balance between levels of vasoconstrictive and vasodilating substances, glycocalyx alterations, and interactions with circulating cells (Figure 1). The crucial issue is to understand which are the major mechanisms that contribute to the microvascular alterations present in septic conditions and, more importantly, whether these could be improved with therapy.

Multiple studies have shown that endothelial dysfunction occurs in sepsis, as evidenced by a decreased sensitivity to vasoconstricting but also vasodilating agents. However, most of these trials used large arteries, up to first-order arterioles, and it is not known to what extent the findings may apply to more distal arterioles and capillaries. In addition, communication between endothelial cells may be altered. Experimentally, Tyml et al. [19] showed that the communication rate between microvessels 500 microns apart was markedly impaired. The study of postischemic hyperemia provides some indirect evidence that endothelial dysfunction may play a role. Using laser Doppler and NIRS techniques, several authors have reported that the postischemic hyperemic response is blunted in patients with sepsis and that these alterations are related to the severity of organ dysfunction [20] and outcome [21].

The interaction between the endothelial surface and circulating cells also is impaired in sepsis. First, the size of the glycocalyx is markedly decreased [22]. The glycocalyx is a layer of glucosaminoglycans that covers the endothelial surface and in which various substances, such as superoxide dismutase and antithrombin, are embedded. The glycocalyx facilitates the flow of red blood cells and limits adhesion of white blood cells and platelets to the endothelium. Interestingly, destruction of the glycocalyx layer by hyaluronidase can mimic sepsis-induced microcirculatory alterations [23].

Activation of coagulation may play a key role in the pathogenesis of microcirculatory alterations [5,24]. In mice challenged with endotoxin, fibrin deposition occurred in a significant proportion of capillaries; the addition of anticoagulant factor decreased the number of nonperfused capillaries, whereas the number was increased after the addition of procoagulant factors [5]. However, microthrombi formation is infrequently observed in experimental sepsis [4].

Finally, circulating cells have a key role in these alterations. Leukocyte rolling and adhesion to the endothelial surface is increased in sepsis [4]. Importantly, this does not only occur at the venular but also at the capillary level [5]. In addition to locally contributing to further activation of the coagulation and inflammatory cascades, the presence of sticking or rolling leukocytes impairs the circulation of other cells. Administration of selectins decreased the adhesion and rolling of white blood cells and improved microvascular perfusion [5]. Adhesion and rolling of platelets also contributes to microcirculatory alterations [4,5]. Finally, red blood cells can contribute to microcirculatory alterations as a consequence of alterations in red blood cell deformability [25], impaired release of nitric oxide, and/or adhesion of red blood cells to the endothelium [26].

### Potential therapeutic interventions

It is crucial to understand that, given the heterogeneous nature of the microvascular alterations, it is more important to recruit the microcirculation than to increase total flow to the organ. Ideally, the intervention should affect one or several of the mechanisms involved in the development of these microvascular alterations. Nevertheless, most interventions that are currently used for their impact on systemic hemodynamics also may somewhat influence the microcirculation.

### Interventions used to manipulate systemic hemodynamics

Fluids and vasoactive agents are key components of hemodynamic resuscitation, with the goal of improving tissue perfusion. However, improved cellular oxygen supply implies an improvement in microvascular perfusion. Two recent trials have demonstrated that fluids can improve microvascular perfusion, increasing the proportion of perfused capillaries and decreasing perfusion heterogeneity [27,28]. Importantly, in both trials the microcirculatory effects were relatively independent of the systemic effects. The microcirculatory effects of fluids seem to be mostly present in the early phase of sepsis (within 24 h of diagnosis), whereas later (after 48 h) fluid administration failed to improve the microcirculation even when cardiac output increased [27]. Whether different types of fluid result in different microvascular responses is still debated. In some experimental conditions, colloids may increase microcirculatory perfusion more than crystalloids [29], but this difference has not been confirmed in septic patients [27]. The mechanisms by which fluids may improve the microcirculation are not well understood but may be related to a decrease in viscosity, to a decrease in white blood cell adhesion and rolling, or, indirectly, to a decrease in endogenous vasoconstrictive substances. Whether the effects of fluids, when observed, will persist or be transient, and also whether this effect can be "saturable," i.e., only the initial effects would be beneficial while further administration of fluids would have minimal effect, requires further study. This "saturable" effect is suggested by the observations of Pottecher et al. [28] who reported that the first bolus of fluids improved microvascular perfusion, whereas the second had no effect even though cardiac output increased further.

The effects of red blood cell transfusions also seem to be quite variable. In one trial, although the effects in the entire population were negligible, transfusions did improve microvascular perfusion in patients with the most severely altered microcirculation at baseline [30].

Beta-adrenergic agents have been shown to improve microvascular perfusion, increasing not only convective but also diffusive transport [31,32]. These effects were dissociated form the systemic effects of these agents [31]. Because capillaries do not have beta-adrenergic receptors, these effects may be mediated by a decrease in white blood cell adhesion, as beta-adrenergic receptors are present on the surface of white blood cells.

Vasopressor agents also have variable effects. Correction of severe hypotension does not impair and may even improve microvascular perfusion [33,34] probably through the restoration of the perfusion of the organs through achievement of a minimal perfusion pressure. However, increasing blood pressure further (mean arterial pressure from 65 to 75 and 85 mmHg) may fail to improve microvascular perfusion. Of note, these data were obtained in small cohort of patients and individual response was provided a huge interindividual variability was observed [35,36]. Interestingly, the increase in arterial pressure impaired the sublingual microcirculation in patients with close to normal microcirculation at baseline, whereas it was beneficial in the most severe cases [36].

Altogether, these data suggest that classical hemodynamic interventions have variable effects on microvascular alterations in sepsis and that these effects cannot be predicted from the evolution of systemic hemodynamics. Often these alterations persist after systemic hemodynamic optimization.

### Other agents

Many other agents have been tested, especially in experimental conditions. We will discuss the effects of some of these agents, which either illustrate the implications of specific mechanisms affecting the microcirculation or have promising effects.

#### Vasodilators

Because local constriction-dilation is implicated in the regulation of flow and capillary recruitment and because decreased vascular density and stopped-flow capillaries may be the result of excessive vasoconstriction, vasodilating substances may have a role in manipulation of the microcirculation. In patients with severe sepsis who have severe microvascular alterations, we demonstrated that topical administration of a large dose of acetylcholine, an endothelium-dependent vasodilating agent, restored the microcirculation to a state similar to that of healthy volunteers and nonseptic ICU patients [2]. This observation has profound implications. First, sepsis-associated microcirculatory alterations are functional and can be totally reversed. Complete obstruction of microvessels by clots is thus unlikely. Second, the endothelium may be dysfunctional but is still able to respond to supraphysiological stimulation. An important limitation of this finding was that we were unable to ensure that excessive vasodilation did not occur, leading to unnecessary high perfusion to some areas with low metabolic rate. As the agent was applied topically, perfusion pressure to the organ was preserved; systemic administration of vasodilating agents may not have the same effects.

In a small series of patients, Spronk et al. [37] reported in a research letter that nitroglycerin administration rapidly improved the microcirculation. These results were challenged by a randomized trial that included 70 patients with septic shock [38] and failed to show any difference in the evolution of the microcirculation with nitroglycerin compared to placebo. Does this second trial close the issue? Probably not, as essential differences exist between the studies. In particular, Spronk et al. [37] assessed the microcirculation 2 min after administration of a bolus dose of 0.5 mg of nitroglycerin while Boerma et al. [38] evaluated the microcirculation 30 min after initiation of a continuous infusion of 4 mg/h (0.07 mg/min). Dosing may be crucial, as illustrated in cardiogenic shock [39], but one should not neglect the fact that these effects may be very transient. Finally, one should note that the microcirculation was minimally altered at baseline in the trial by Boerma et al. [38], as the proportion of perfused capillaries was already normal (98%), leaving no room for further improvement. Other vasodilating agents have been used, especially in experimental models. Salgado et al. [40] recently evaluated the effects of angiotensin converting enzyme inhibition in an ovine model of septic shock. The sublingual microcirculation was slightly less severely altered in treated animals compared with controls, but these effects were not accompanied by an improvement in organ function. Accordingly, at this stage, the use of vasodilating agents cannot be recommended. One of the reasons for this relative failure is the lack of selectivity of these agents, which dilate both perfused and nonperfused vessels, thereby possibly leading to luxury perfusion of some areas.

#### Anticoagulant agents

Activated protein C has repeatedly been shown to improve the microcirculation in different experimental models and in various organs [22,41,42]. Similar results were observed in a controlled but not randomized trial, which showed that the sublingual microcirculation improved already 4 hours after initiation of therapy, whereas it remained stable in controls [43]. Similar beneficial results were observed with antithrombin in experimental conditions [44]. The anticoagulant effect seems not to be involved in the microcirculatory effects of these agents. Indeed a modified antithrombin, deprived of its ligation site for the endothelium but with preserved anticoagulant activity, failed to improve the microcirculation in endotoxic animals [44]. In addition, hirudin, a pure thrombin inhibitor, did not improve the microcirculation of septic animals [45]. What then could be the mechanisms involved in the beneficial effects of these agents? Decreased white blood cell and platelet rolling and adhesion [41,42], preservation of glycocalyx size [22], and improvement in endothelial reactivity [46] are the most likely mechanisms.

#### Steroids

Hydrocortisone is frequently used as an adjunctive therapy in patients with septic shock. Hydrocortisone facilitates weaning of vasopressor agents. Hydrocortisone may induce some degree of arteriolar vasoconstriction and this could alter capillary perfusion. It also may improve endothelial function and thereby ameliorate the distributive defect. In healthy volunteers in whom endothelial venular dilation is impaired by local cytokine infusion, hydrocortisone administration can rapidly reverse this phenomenon [47]. In 20 patients with septic shock, Buchele et al. [48] observed that hydrocortisone improved microvascular perfusion slightly. This effect was already observed 1 hour after hydrocortisone administration and persisted during the entire observation period. Interestingly, these effects were independent of any change in arterial pressure.

Among the proposed mechanisms, steroids may improve endothelial function [47], preserve the glycocalyx [49], or decrease rolling and adhesion of white blood cells to the endothelium [50].

#### Vitamin C and tetrahydrobiopterin

Vitamin C and tetrahydrobiopterin have many important actions, including the correct function of endothelial nitric oxide synthase. Deficiency of both these substances may occur in sepsis. In rodents, administration of vitamin C improved microcirculatory perfusion, increasing capillary density and decreasing the number of stopped flow capillaries [9,51]. Importantly these beneficial results persisted even when vitamin C was administered up to 24 h after the initiation of sepsis [9]. Similar beneficial effects have been observed with tetrahydrobiopterin [5,51]. These promising results need to be confirmed in large animal models and in humans.

## Conclusions

Multiple experimental and clinical trials have shown that microcirculatory alterations occur in sepsis and that these may play a role in the development of organ dysfunction. These alterations are characterized by a decrease in capillary density and in heterogeneity of capillary perfusion with stopped-flow capillaries in close vicinity to well-perfused capillaries. Various mechanisms can be implicated in the development of these alterations, including endothelial dysfunction and failure of communication between endothelial cells, glycocalyx alterations, and altered interactions between the endothelium and circulating cells. Given the heterogeneous aspect of microcirculatory perfusion and the mechanisms involved in the development of these alterations, it is expected that classical hemodynamic interventions will only minimally affect the microcirculation. Vasodilating agents have been suggested to influence the microcirculation, but their administration may be limited by the risk of hypotension and their lack of selectivity, potentially leading to luxury perfusion. Other interventions are currently in the pipeline, most of these aimed at modulating endothelial function.

## Competing interests

Daniel De Backer and Jean-Louis Vincent have received honoraria for lectures and research grants from Eli Lilly. The other authors declare that they have no competing interests.

## Authors' contributions

DDB drafted the manuscript. The manuscript was revised for important intellectual content by KD, FST, GOT, DS, and JLV. All authors read and approved the final manuscript.
